# Agricultural Microbes Genome 2

**DOI:** 10.1002/cfg.64

**Published:** 2001-02

**Authors:** Peter J. Johnson, Noel T. Keen, Joan K. Lunney, Michael J. Sadowsky

**Affiliations:** 1 USDA-CSREES-National Research Initiative First class mail: Stop 2241, 1400 Independence Avenue SW Washington DC 20250-2241 USA; 2 Plant Pathology Department University of California at Riverside Riverside CA 92521 USA; 3 Immunology and Disease Resistance Laboratory ANRI, ARS, USDA Building 1040, Room 107 Beltsville MD 20705 USA; 4 Department of Soil, Water, and Climate, and Biological Process Technology Institute University of Minnesota 1991 Upper Buford Circle 439 Borlaug Hall St. Paul MN 55108 USA; 5 USDA-CSREES-National Research Initiative Express mail: Room 2454, 800 9th Street SW Washington DC 20024 USA


**Comparative and Functional Genomics**
*Comp Funct Genom* 2001; **2**: 10–13.

**Conference Editorial**

### Agricultural Microbes Genome 2

Peter J. Johnson[1]*, Noel T. Keen[2], Joan K. Lunney[3] and Michael J. Sadowsky[4]

[1] *USDA-CSREES-National Research Initiative, Stop 2241, 1400 Independence Avenue SW, Washington, DC 20250-2241, USA*
[2] *Plant Pathology Department, University of California at Riverside, Riverside, CA 92521, USA*
[3] *Immunology and Disease Resistance Laboratory, ANRI, ARS, USDA, Building 1040, Room 107, Beltsville, MD 20705, USA*
[4] *Department of Soil, Water, and Climate, and Biological Process Technology Institute, University of Minnesota, 1991 Upper Buford Circle, 439 Borlaug Hall, St. Paul, MN 55108, USA*

**Correspondence to:*
*P. Johnson, USDA-CSREES-National Research Initiative. Express mail: Room 2454, 800 9th Street SW, Washington, DC 20024, USA. First class mail: Stop 2241, 1400 Independence Avenue SW, Washington, DC 20250-2241, USA.*
*E-mail: PJohnson@reeusda.gov*

### Overview

Agriculture has a major impact on the well-being of this planet and is imperative for the sustainability of the world's population. In the USA alone, agriculture generates nearly $1 trillion annually or 13% of the gross national product. Knowledge of the genomes of agricultural microorganisms is expected to underpin future advances in agriculture into the next quarter century. Genomics will serve as the driving force for research in the life sciences, including agriculture, the environment and food safety. An accelerated understanding of beneficial and pathogenic microorganisms will lead to more rapid advances in metabolic engineering, the development of sensitive and specific diagnostic tools, the marketing of improved therapeutics and efficacious vaccines, and the conversion of agricultural materials into high-value products such as fuels and chemicals.

Although several microbial genomics conferences had become well established prior to 2000, none included a major focus on microbes relevant to agriculture. To foster coordination and information exchange for microbial genomics among the world wide agricultural community, the USDA co-sponsored the first International Agricultural Microbes Genome (AMG1) Conference in January 2000. The conference highlighted advances relative to sequencing, bioinformatics and functional genomics for microbes relevant to the plant, animal and natural resource areas. Several milestones were announced, including: the sequencing, by a consortium of European researchers, of *Listeria monocytogenes*, an important animal health and food safety pathogen; a Brazilian team's sequencing of the first bacterial plant pathogen, *Xylella fastidiosa*; and the impending annotation of an avian strain of *Pasteurella multocida* at a US institution. Nineteen countries were represented at this inaugural meeting, with an attendance that exceeded 160 scientists and policy makers.

Based on feedback from AMG1 participants, the scope and length of the Agricultural Microbes Genome 2 Conference was expanded. Three plenary sessions included sequencing, technology and bioinformatics, and functional genomics and applications. Three workshops covered bioinformatics, funding opportunities, and presentations for USDA-sponsored genomics projects.

The USDA intends to continue support for the Agricultural Microbes Genome Conference (**http://www.intl-pag.org**) so that relationships among scientists involved in different areas of agriculture continue to mature. With the increasing number of

Copyright © 2001 John Wiley & Sons, Ltd.

microbial genomics conferences that have emerged, it may also be time to pursue opportunities to coordinate some efforts. Issues related to new technologies, database
connectivity, informatics, training, outreach and access to resources are common concerns, regardless of one's primary mission area. As stronger linkages develop among the fields of agriculture, human health, energy production, bioremediation and other areas engaged in genomics, there will be a better capitalization of opportunities to the benefit of all. Further integration that increases the critical mass of genomics expertise will be a win–win situation for all areas and disciplines.

### Sequencing session (Chair: Noel T. Keen)

Following announcement of the *Haemophilus influenzae* genomic sequence by C. Fraser and colleagues at The Institute for Genomic Research (TIGR), more than 50 microbial genome sequences have been announced or are in progress. Unfortunately, few of these have thus far been derived from agriculturally important microorganisms—this despite the substantial economic and social impacts of such organisms. Fortunately, the previous oblivion that characterized granting agencies serving this research field seems to be abating and sequencing projects are now proceeding or are ready to be initiated on a sizeable number of agriculturally important microbes.

As the number of agriculturally important microorganisms that are sequenced increases, we can expect major payback in the form of improved manipulation of these microbes to promote human welfare. For example, disease-causing organisms in plants and animals will be more amenable to the development of new control measures if their entire genome sequences are known. In addition, it is a good bet that microbial genome sequences, especially those from microbes with unique niches such as these, will yield genes encoding unique protein products that may have great scientific and societal value.

AMG2 will announce the genomic sequencing of several agriculturally important microbes. The session will be initiated by Claire Fraser from TIGR, discussing the utility of genome sequencing in studies of physiology and evolution. Claire will bring to bear her long-standing experience and insight with microbial genome analyses and, in the process, will outline a blueprint for the future.

Several speakers will address genome sequencing projects on agriculturally important microorganisms. Fernando Reinach from the University of Sao Paulo will introduce the complete sequence of *Xanthomonas citri* (**http://watson.fapesp.br/xantho/main.htm**). This is an economically important pathogen of citrus and is a member of a large group of plant pathogenic bacteria that are specialized to attack particular plant species. The disease caused by *X. citri*, for example, is currently wreaking havoc in the US state of Florida.

Chris Minion of Iowa State University will discuss what has been learned from the complete genome sequence of an important animal pathogen, *Mycoplasma hyopneumoniae* (**http://mycoplasmas.vm.iastate.edu/seq/home.html**). He will also discuss problems encountered during the sequencing process of this small genome.

Stanley Maloy, University of Illinois, will discuss the work of his group with several *Salmonella* serovars of animal hosts (**http://www.life.uiuc.edu/micro/maloy.html**). It is known that these bacteria have common core virulence genes as well as sets of unique genes that define virulence on particular animal hosts.

Christian Boucher of INRA in France will describe the genome sequencing project with *Ralstonia solanacearum* (**http://www.toulouse.inra.fr/lbmrpm/eng/hp_cb.htm**), a soil-borne plant pathogen that is quite destructive and has a wide host range. In addition to permitting the identification of new candidate genes for pathogenicity, this project reveals important features concerning the structural organization of the bacterial genome.

### Technology and bioinformatics session (Chair: Joan K. Lunney)

This session explores the major developments in speed and capacity of genome sequencing and gene expression studies and the impressive new capacities of bioinformatics resources. Tom Slezak, Lawrence Livermore National Laboratory, will discuss the US Department of Energy's (DOE) microbial genome marathon. During October 2000, high-quality draft sequences of 15 bacterial genomes were produced

Copyright © 2001 John Wiley & Sons, Ltd. 

at the Joint Genome Institute (**http://www.jgi.doe.gov/**). Included in this, their first 'Microbial Month', was *Xylella fastidiosa*, the pathogen carried by sharpshooter insects that infect grapevines. Principal biological researchers will be invited to an annotation jamboree in the next few months. A similar 'Ag Microbial Month' is certainly a possibility!

The astonishing increase in sequencing capacity is mirrored by the increase in publicly available bioinformatics tools for genome comparisons. Michael Galperin, NIH/NLM/NCBI, will review the Clusters of Orthologous Groups of proteins (COGs). These were delineated by comparing protein sequences encoded in 30 complete genomes, representing 26 major phylogenetic lineages. Each COG consists of individual proteins or groups of paralogues from at least three lineages and thus corresponds to an ancient conserved domain (**http://www.ncbi.nlm.nih.gov/COG/**). Advances in defining complementary genome patterns with COGs assists in annotating analogous forms of genes. Unusual gene patterns point to potential new drug targets or, sometimes, to incomplete genome sequencing.

Gene expression data is rapidly transforming our understanding of genomics. Gene expression studies elucidate potentially co-regulated genes. Terry Gaasterland, Rockefeller University, will discuss bi-directional analyses, using comparative genome annotations and gene expression data, as a first step toward enabling users to evaluate the gene expression patterns of molecular subsystems. She has prototyped the method as the 'Cluster Explorer' module of the TANGO (Transcriptome Analysis of Genomes) system. TANGO is integrated with the MAGPIE genome sequence annotation system (**http://genomes.rockefeller.edu/research.shtml#magpie**).

As microbial genomics advances the new frontier is proteomics. Michael Kertesz, University of Manchester, has studied *Pseudomonas aeruginosa,* a versatile Gram-negative species that grows in soils and sediments, as well as being a pathogen of plants and humans. It was the first *Pseudomonas* species to be targeted for genome sequencing (**http://www.pseudomonas.com/**). Proteomics of the response of *P. aeruginosa* to sulphate starvation will be presented using differential two-dimensional PAGE followed by Edman N-terminal sequencing and MS sequencing (MS–MS). After gene identification, reverse-transcription PCR (RT–PCR) was used to confirm that repression in the presence of sulphate was occurring at a transcriptional level. His study demonstrates the power of a combined proteomic/genomic/transcriptional analysis approach for investigation of the responses of an organism to an environmental stimulus.

### Functional genomics and applications session (Chair: Michael J. Sadowsky)

This session examines both functional aspects and applications of microbial genomics. Microbial genomics is proving to be a fast-moving and exciting field of science. While microbial genome sequencing has largely focused on the Archaea and those of importance to human health and medicine, bacteria of consequence to the environment and agriculture have largely been ignored. Contrary to scientific expectations, sequence data from the microorganisms sequenced to date show little repetition. Consequently, determination of the function(s) of many microbial genes, based on their relatedness to those in databases, has proven to be a difficult if not a daunting task. Thus, new technologies, including micro- and macroarrays, will no doubt provide a lot of insight into the function(s) of many microbial genes.

Colin Harwood, Sam G. Crawshaw and Anil Wipat from Newcastle University, UK, will discuss the systematic sequencing and functional analysis of *Bacillus subtilis* (**http://www.ncl.ac.uk/dmi/harwood/harwoodwp.html**). While this organism has long been used as a model biological system to study sporulation and novel sigma factor control systems, and the genetics is well understood, little is known about the way in which this soil microorganism interacts with plants and its environment. Since the complete genome sequence of *B. subtilis* was finished in 1997, Dr Harwood and his co-workers, using a number of new and innovative approaches, have begun to identify the function of unknown open reading frames and to understand this organism's global regulatory responses to environmental stress. This will yield insight into the use of the organism for biocontrol purposes and to study rhizosphere colonization at the molecular level.

Many soil microorganisms are involved in the biogeochemical cycling of elements. *Nitrosomonas europaea*, an obligate chemoautolithotroph, plays a pivotal role in the oxidation of ammonia (nitrification) on a global scale. Up until this date, there is little genomic information concerning the

Copyright © 2001 John Wiley & Sons, Ltd.

chemolithotrophic $\beta$-Proteobacteria. Dan Arp (Oregon State University) and colleagues at the Joint Genome Institute (**http://www.jgi.doe.gov**) have sequenced this organism to 8X coverage. Sequencing results have revealed that, of 2200 genes present, only about 1200 can be identified based on similarity searches. While all genes for central biosynthetic pathways are essentially complete and include genes for synthesis of amino acids, nucleotides, co-factors, sugars and lipids, many catabolic pathways are strikingly absent, indicating that this organism is 'locked' into a chemolithotrophic existence. Interestingly, despite the fact that sequencing results indicate that ammonia assimilation may occur by glutamate dehydrogenase or GS/GOGAT, the genetic basis for obligate autotrophy remains unclear. The sequences from this interesting microorganism can be found at **http://spider.jgi-psf.org/JGI_microbial/html/nitrosomonas_homepage.html.**

Although genomic sequencing of bacteria and Archaea are proceeding at a rapid pace, less is known about the genomics of Protozoa. *Cryptosporidium parvum* is a well-recognized cause of diarrhea in humans and animals throughout the world. The infective stage of this obligate, intracellular, protozoan parasite, the oocyst, is about half the size of a red blood cell and sporocysts are resistant to many chemical disinfectants. However, little is known about its virulence factors, genome structure and gene regulation that contribute to infection. To address these issues, Mitch Abrahamsen and colleagues at the University of Minnesota have undertaken the task of sequencing the entire *Cryptosporidium parvum* genome (**http://www.cbc.umn.edu/ResearchProjects/AGAC/Cp/index.html**). The *C. parvum* genome is estimated to be about 10.4 Mb in size and sequencing to a seven-fold coverage is being done using shotgun cloning in double-stranded plasmid vectors. Currently, an approximately two-fold coverage of the genome has been completed, resulting in assembled contigs representing $>70\%$ of the genome. Their study is important, as it will provide an understanding of the biology of this obligate, intracellular parasite and information that can be potentially used in disease prevention programmes.

*Sinorhizobium meliloti* (formerly classified as *Rhizobium meliloti*) is a soil bacterium that enters into a symbiotic nitrogen-fixing symbiosis with leguminous plants of the genera *Medicago.* This member of the alpha subdivision of Proteobacteria possesses a complex genome which contains three replicons: a 3.5 Mb chromosome and two mega-plasmids, pSymA (1.4 Mb) and pSymB (1.7 Mb). The majority of genes required for symbiosis are located on all three replicons. Sharon Long at Stanford University (**http://cmgm.stanford.edu/~mbarnett/genome.html**) and members of an international consortium have undertaken the effort to sequence the entire genome of *S. meliloti* strain 1021. The completion date for this project is estimated for Fall 2000. In addition to a 1X random pass of the total genome, her laboratory plans to contribute to the international sequencing project by obtaining the complete sequence of the pSymA replicon. Information obtained from this sequencing and functional genomics project will provide for a more complete understanding of symbiosis-related genes in this and related micro-organisms, and yield insight into the complex signalling pathways involved in plant–microbe communication.

Copyright © 2001 John Wiley & Sons, Ltd.

